# Hypothalamic–pituitary–adrenal axis in anorexia nervosa; an underestimated endocrine dysfunction among adolescents

**DOI:** 10.3389/fped.2024.1415061

**Published:** 2024-12-12

**Authors:** Valeria Calcaterra, Vittoria Carlotta Magenes, Nadia Fratangeli, Giulia Nigro, Valentina Fabiano, Leonardo Mendolicchio, Gianvincenzo Zuccotti

**Affiliations:** ^1^ Pediatric and Adolescent Unit, Department of Internal Medicine, University of Pavia, Pavia, Italy; ^2^ Pediatric Department, Buzzi Children’s Hospital, Milan, Italy; ^3^ Experimental Laboratory for Metabolic Neurosciences Research, I.R.C.C.S. Istituto Auxologico Italiano, Piancavallo, Italy; ^4^ Department of Biomedical and Clinical Science, University of Milan, Milan, Italy

**Keywords:** anorexia nervosa, hypothalamic-pituitary-adrenal, cortisol, endocrine dysfunction, adolescents

## Abstract

In patients affected by anorexia nervosa (AN) different endocrine abnormalities have been described, but, among them, hypothalamic–pituitary–adrenal (HPA) dysfunction, although associated to important side effects, is underestimated and has no therapeutical options. We present a narrative literature review to investigate the HPA axis in patients with AN, in order to highlight HPA dysfunction and its effects. We also described the crucial role of HPA monitoring, and to consider eventual therapeutic and preventive strategies in AN patients. The literature now available demonstrates that women and girls suffering from AN have higher measures of cortisol and lower levels of androgens as compared to controls. These endocrinological disturbances have deleterious effects on the subjects, both from the physical and from the psychological point of view. It's fundamental for physicians to consider these aspects when assessing AN patients. The mechanisms behind the adrenocortical dysfunctions in eating disorders patients remain an open question and there are no available treatments, thus research on this issue would be extremely useful and highly necessary, especially in the pediatric field.

## 1 Introduction

Eating disorders are one of the most prevalent chronic disorders in adolescents and young adults, with an important prevalence in younger children (1, 2). Among these disorders, anorexia nervosa (AN) is characterized by altered body image, persistent food restriction and resultant low body weight (3). This condition has a prevalence of at least 0.2%–1% among adolescent girls and young women (1–3).

Nutritional and hormonal factors are closely related to the regulation of metabolism (4) and nutritional alterations have been shown to affect the functioning of the endocrine glands, possibly leading to serious disorders (5).

In patients affected by AN different endocrine abnormalities have been described, but, among them, hypothalamic–pituitary–adrenal (HPA) dysfunction, although associated to important side effects, is underestimated and has no therapeutical options.

Common comorbidities of AN include electrolyte abnormalities, anaemia, amenorrhea, and bone demineralization (3, 4). Many of these medical complications can be attributed to hormonal unbalances due to chronic undernutrition and the significant physiologic stress correlated to it.

Indeed, physiologic stress activates the HPA axis and several studies have reported higher cortisol levels in patients with AN, a clear example of chronic undernutrition and persistent stress Cortisol level play an important role in the association of malnutrition with chronic diseases, sustaining a vicious cycle comprised of insufficient food intake and disease.

In addition to cortisol, AN patients tend to have unbalanced levels of others hormones produced by the adrenal cortex such as dehydroepiandrosterone, dehydroepiandrosterone-sulphate, progesterone, aldosterone, testosterone, and other androgens, with consequent complications both from the psychological and from the physical point of view (4, 6, 7). Additionally, a role of HPA axis disturbances on the other hormonal disorders, including thyroid function, could also be considered in AN.

Understanding the mechanisms responsible for endocrine complications of AN is important given the severity and chronic nature of the disease and its complications.

Hence, this narrative review aims to delve deeper into the HPA axis in patients with AN with a specific focus on adolescents. The objective is to illuminate the dysfunction of the HPA axis and its implications, including mood disturbances and physical adverse effects such as estrogen deficiency and bone demineralization. Moreover, our work aims to describe the crucial role of HPA monitoring, and to consider eventual therapeutic and preventive strategies in AN patients.

## 2 Methods

We present a narrative literature review assessing hypothalamic–pituitary–adrenal axis dysfunction and its implications in patients with AN. Reviews, original research papers, meta-analyses, clinical trials, and case reports published in the last 10 years, up to February 2024 were examined. Letters, commentaries, and articles without full text available in English were excluded. We conducted a non-systematic search on the PubMed, Scopus, and Web of Science platforms. The following terms (alone and/or in combination) were used for the literature search: eating disorders, AN, undernutrition, endocrine, hypothalamic–pituitary–adrenal axis, cortisol, androgens, mood disturbances, physical adverse effects, bone mineralization, menstrual disorders. The selection was performed considering all the most significant data in adult and pediatric age; subsequently we focused the discussion on the adolescents. Starting with a total of 128 papers the authors assessed the abstracts (*n* = 105) and reviewed the full texts of relevant articles (*n* = 80) that were analyzed and discussed. Furthermore, we checked the references of all reviewed articles.

**Figure 1:**
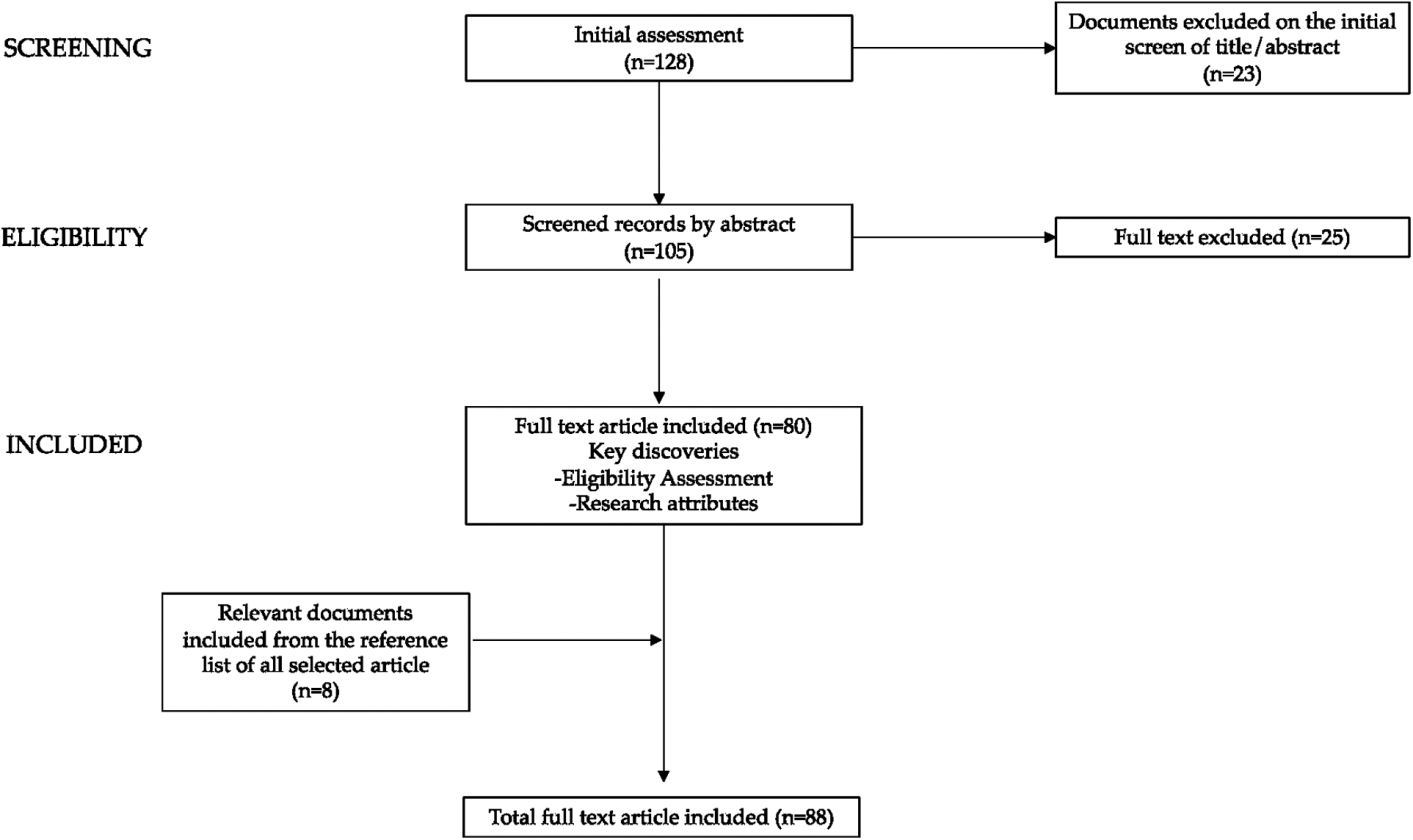
Flow diagram of paper selection and exclusion is presented graphically.

In Figure 1, the diagram depicting the process of paper selection and exclusion is presented graphically.

## 3 Anorexia Nervosa: definition and epidemiology in children/adolescents

### 3.1 Definition

AN is a mental health disorder, being part of FED (feeding—eating – disorders), defined by the recent revision of “*Diagnostic and Statistic Manual of Mental Disorders*” DSM-5 by three main criteria:

(1) significantly low body weight meant as “Restriction of energy intake relative to requirements, leading to a significant low body weight in the context of the age, sex, developmental trajectory, and physical health” (8)
(2) fear of weight gain and/or dysfunctional behaviors aimed at weight control meant as “Intense fear of gaining weight or becoming fat or persistent behavior that interferes with weight gain” (8)
(3) loss/distorted perception of body image (body image distortion) meant as “Disturbed by one's body weight or shape, self-worth influenced by body weight or shape, or persistent lack of recognition of seriousness of low bodyweight” (8)

DSM-5 also identifies 2 subcategories:

(4) **restrictive anorexia**: the weight loss, in the last 3 months, is obtained with restrictive diets, or reduction in intake or physical hyperactivity (8).
(5) **atypical anorexia**: binges followed by food restrictions, abuse of laxatives, self-induced vomiting, sometimes without evidence of pathological BMI (8). In cases of atypical AN, often the individual's weight is within or above the normal weight range (9).

Differently from DSM IV, in the fifth edition of the Diagnostic and Statistical Manual of Mental Disorders, (published by the American Psychiatric Association in 2013) items as “refusal to maintain body weight at or above a minimally normal weight for age and height” or a “denial of the seriousness of low body weight” (implying a deliberate attitude of the patient and willful actions) have been dropped in and replaced by more neutral terms, such as “restriction of energy intake relative to requirements” and “persistent lack of recognition of the seriousness of the current low body weight”.

These same changes gave more prominence to the modifications that occur during developmental age, allowing the changes to be evaluated the context of “age, sex, developmental trajectory and physical health”, even if at least the clinicians suffer by the lack of a standard or reference for the weight criterion, especially in the younger patient groups. According to DSM-5, significantly low body weight in children and adolescents is defined as “weight that is less than minimally expected”.

For adults, a BMI ≤ 18.5 kg/m^2^ is established to be a cut off of underweight according to both the Centers for Disease Control and Prevention (CDC) and the World Health Organization (WHO) (10).

For childhood and adolescent patients BMI-centiles were introduced to allow comparability of body weight adjusted for height across childhood and adolescence so that DSM-5 refers to the CDC definition of underweight based on a “BMI-for-age <5° centile” “that is not due to another health condition or the unavailability of food” (10).

In pediatric-adolescent population, the severity degree of AN is defined by ICD-11 as a BMI below the fifth centile and ≥0.3rd centile and a BMI <0.3rd centile for the specification in two subcategories: significantly and dangerously low body weight respectively (11).

In 2019 Engelhardt et al. reported how, since no correlation to age was demonstrated in younger patients BMI-SDS, a specific BMI-SDS or BMI-centile (e.g., 10th BMI-centile; see below) could be used as the weight criterion (12), especially under age of 15 yrs.

In addition the DSM-IV criteria for AN included amenorrhea, or the absence of menstrual cycles, as a required symptom for diagnosis in females. In contrast, the DSM-5 criteria removed this requirement, recognizing that individuals of all genders can exhibit AN symptoms without necessarily experiencing amenorrhea, thus broadening the diagnostic criteria to be more inclusive (8).

### 3.2 Epidemiology

The literature presents conflicting data on the incidence and prevalence of anorexia nervosa (AN) in childhood. Some studies indicate an increase in recent years (13), others do not confirm this trend (14, 15).

The prevalence of eating disorders may be underestimated for several reasons: not all types of eating disorders are included in many epidemiological surveys, leading to the assumption that prevalence rates might be significantly higher. Additionally, the lack of global epidemiological data, variations in assessment methods, and inconsistencies among studies limit the comparability of findings, especially given that few researchers use the new DSM-5 diagnostic criteria.

The overall distribution varies widely in different geographical areas: the incidence is greater in western countries (Europe, US, China, Japan) and very low in some other areas. These items underline how socio-cultural factors can impact the development of AN (16).

To date, few studies used DSM-5 to investigate the prevalence of eating disorders, but they demonstrated an increased rate of AN in children and adolescents (despite the frequency of disorders reported according to DSM-IV) (17). An increase was also found in epidemiologic studies when comparing DSM-IV prevalence rates with the prevalence rates obtained according to DSM-5 (18, 19).

Thus, studies that use DSM-5 diagnostic criteria to conduct comprehensive investigations of all types of eating disorders are still lacking. The prevalence of AN using the diagnostic criteria of DSM-5 was much higher than that using DSM-IV, in accord with other study results (20–22). The major studies evaluating the epidemiological trends of this pathology are reported in Table 1.

**Table 1:** Relevant studies assessing epidemiological trends in eating disorders.

Reference	Geographical area	Years evaluated	Incidence/prevalence of EDs	Criteria used
Currin et al. (23)	England	1970s–2000s	Increase in EDs incidence; estimated incidence of AN ranged between 4.7 to 7.7 per 100,000 people.	DSM-IV
Machado et al. (24)	Four geographical areas of Portugal (North, Centre, Lisbon Metropolitan Area, and South)	–	Prevalence of EDNOS: 40% of diagnosed eating disorder cases in various community samples.	DSM-IV
Stice et al. (25)	United States	8 years, 2000s	Lifetime prevalence of AN by age 20: 0.8%; combined prevalence of threshold, subthreshold, and partial EDs.	DSM-IV
Qian et al. (26).	Global	2013–2021	Pooled lifetime prevalence of EDs at 0.91% (95% CI, 0.48–1.71); higher prevalence in Western countries; pooled lifetime prevalence of AN using DSM-5 was 0.89%, ∼9 times higher than DSM-IV.	DSM-IV vs. DSM-5
Silvestri et al. (27)	Italy	Health register up to 1st January 2019.	Preliminary report on mental disorder prevalence in an Italian region; estimated prevalence of AN around 0.3%–0.4%.	DSM5

AN, anorexia nervosa; EDs, eating disorders; EDNOS, eating disorders not otherwise specified; CI, confidence interval; DSM, diagnostic and statistical manual.

The age of onset of AN has progressively decreased, even if is not possible to estimate the exact prevalence and incidence in different age groups, due to studies that pool data from childhood with adolescent age.

By the way, it's possible to assert that in the last 2 decades in Europe the share of subjects with AN under the age of 15 has increased (in particularly, between 2000 and 2017, the age of onset was below 13 ys) (28–30). This trend was documented in Germany, Portugal and in Italy (28–30). In UK, the overall incidence for eating disorders in 12 to 13 yrs old is estimated about 9.51/100,000, similar to the Canadian one for the 10–12 age group (31).

The ratio of males to females in several studies shows a higher proportion of females affected by AN (30, 32). This percentage could be even related to underdiagnosed and undertreated male children/adolescent with AN.

The incidence and prevalence rates of childhood -onset AN still remain difficult to determine precisely (33) and specific studies on the purely pediatric population are still rare, indeed children under the age of 13 years currently represent a much smaller group than adolescent patients.

## 4 The hypothalamic-pituitary-adrenal axis

The hypothalamic-pituitary-adrenal axis (HPA) is a neuroendocrine system of transduction-amplification of signals from the brain to the periphery and represents the key point of integration between the endocrine, nervous and immune systems. The HPA axis consists of a cascade of endocrine pathways that respond to specific negative feedback loops involving the hypothalamus, anterior pituitary gland, and adrenal gland (34).

Activation of the HPA axis is mediated by neurosecretory neurons located in the medial parvocellular division of the paraventricular nucleus (PVN) which is the major source of corticotropin-releasing hormone (CRH) (35). Arginine vasopressin (AVP), colocalized in a portion of CRH neurons in the parvocellular division of the PVN (36), is incorporated into secretory granules together with CRH and co-secreted into the portal circulation in response to particular stresses, as reported on animal studies (36). AVP is a potent synergistic factor with CRH in stimulating ACTH secretion, but has little ACTH secretagogue activity alone, as reported on human studies (37).

CRH is a 41 amino acid peptide, which increases ACTH secretion and POMC gene expression in corticotrope cells of the anterior pituitary gland, via the G protein-coupled receptor CRH-1; CRH stimulates ACTH according to a circadian rhythm, peaking before awakening progressively declining throughout the day. It is also released in response to stress, whether emotional, physical, or organic overload of the body (38).

Anterior pituitary corticotroph cells produce ACTH by selective proteolytic processing of POMC (39).

The primary function of ACTH is to stimulate the secretion of glucocorticoids and androgens from the adrenal glands, while playing a minor role in regulating mineralocorticoid function. The N-terminal portion of ACTH is responsible for its biological activity. The stimulating action is biphasic: a rapid response in case of stress, through the immediate release of steroids from a ready-release pool; at the same time, a neosynthesis of cholesterol is activated, which can last from a few minutes to a few hours (40).

Foppiani et al. investigated the effects of desmopressin on the release of ACTH and cortisol in response to ovine CRH in individuals with anorexia nervosa and showed that desmopressin did not affect ACTH or cortisol responses to CRH in these patients (41).

The adrenal gland is made of adrenal cortex that is divided into three separate zones (zona glomerulosa, zona fasciculata and zona reticularis), and adrenal medulla.

Cortisol is a glucocorticoid hormone produced by the zona fasciculata that helps control the body's use of fats, proteins and carbohydrates; suppresses inflammation; regulates blood pressure, regulates glycemia and can also decrease bone formation. It is released during times of stress but the levels in the unstressed state vary in a circadian rhythm with peak levels occurring in the 2- to 4 h window around waking up, and nadir levels occurring in a similar time frame around sleep onset (42).

Aldosterone is produced by the zona glomerulosa and plays a central role in regulating blood pressure and electrolytes (sodium and potassium). DHEA and androgenic steroids hormones are produced by the zona reticularis. The adrenal medulla controls hormones that initiate the flight or fight response. The main hormones secreted by the adrenal medulla include epinephrine (adrenaline) and norepinephrine (noradrenaline).

In conditions of chronic stress the effect of ACTH can be observed, with cellular hyperplasia, angiogenic effect and increase in stromal tissue. The chronic effect of ACTH modifies the production of the adrenal cortex, increasing the synthesis of glucocorticoids and reducing androgens and aldosterone (40).

**Figure 2:**
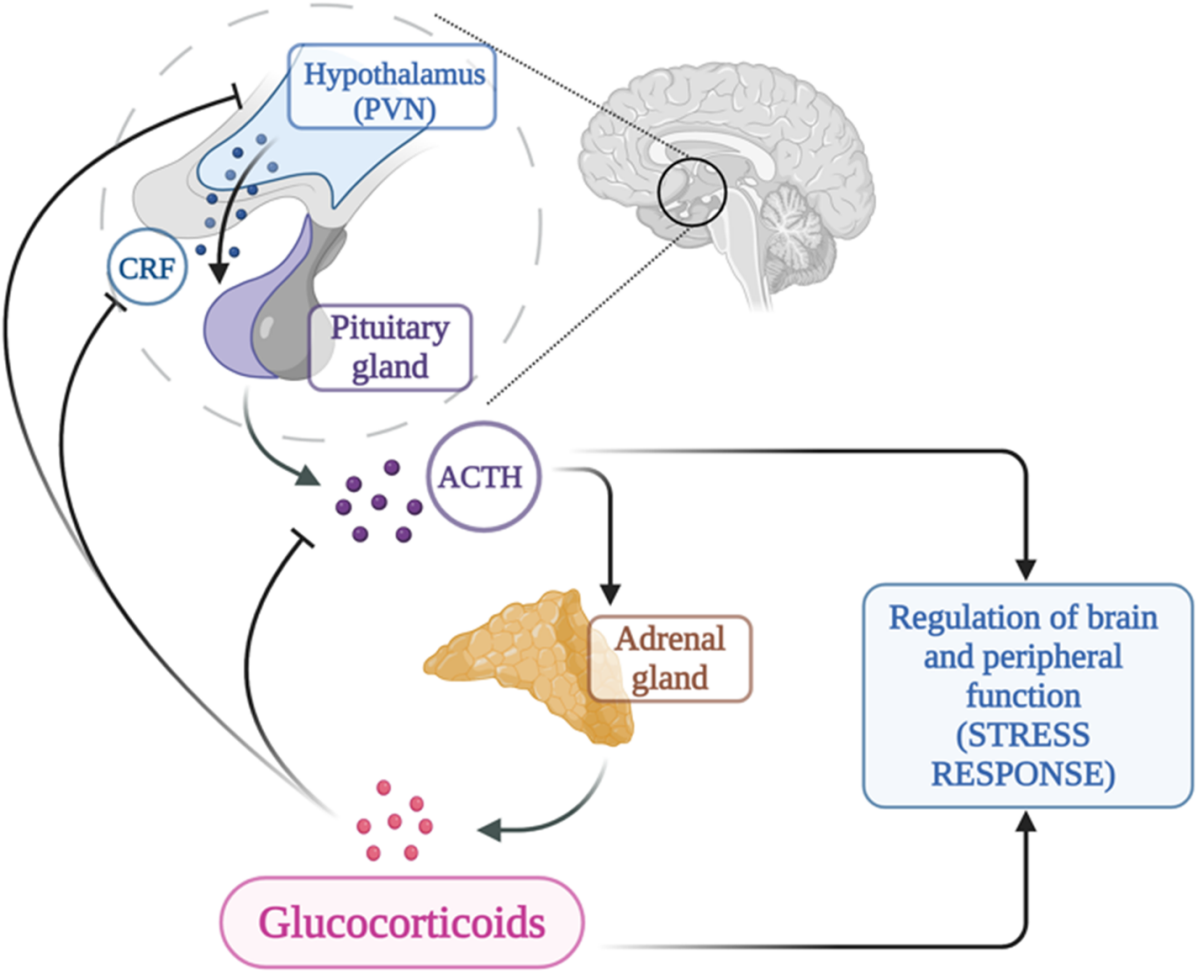
Regulation of the hypothalamo-pituitary adrenal axis (created by biorender®).

The HPA axis is subject to feedback inhibition from circulating glucocorticoids (43), as shown in Figure 2. Glucocorticoids modulate the HPA axis through at least two distinct mechanisms of negative feedback: delayed feedback system that is responsive to glucocorticoid levels and involves genomic alterations and fast nongenomic feedback system that is sensitive to the rate of glucocorticoid secretion; the role of each of these systems and their regulatory mechanisms may provide new therapeutic targets for treatment and prophylaxis of stress-related disorders including anxiety, feeding, addiction, and energy metabolism (43, 44).

## 5 Hypothalamic–pituitary–adrenal axis in AN: hormone levels and consequences of variations

The endocrine changes associated with AN have been studied in depth and provide strong evidence for hypothalamic dysfunctions (4, 6).

With regard to the hypothalamic–pituitary–adrenal axis, different studies showed that in about one-third of patients affected by AN, the HPA axis is in a chronically stimulated state (4, 7, 45–48). It's well known that physiologic stress activates the hypothalamic–pituitary–adrenal axis and indeed in these patients chronic undernutrition causes a significant physiologic stress (6).

**Figure 3:**
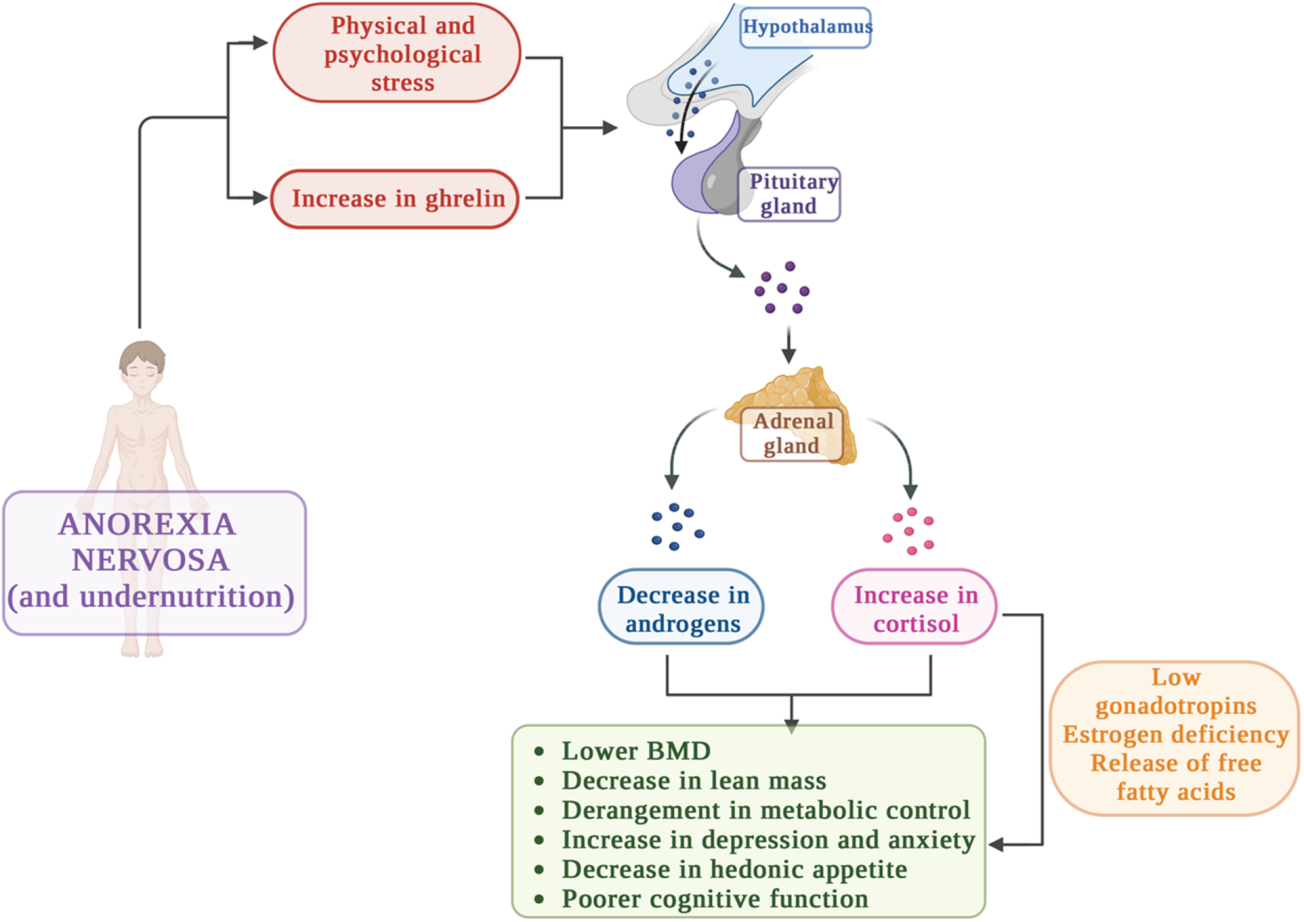
Hypothalamo-pituitary adrenal axis unbalances in AN: causes and consequences.

The main HPA unbalances in AN and the consequences are depicted in Figure 3.

The involvement of the HPA axis has also been demonstrated by brain functional magnetic resonance imaging (MRI) studies, which have reported pituitary atrophy secondary to nutritional or endocrine alterations rather than primary pituitary pathology (49) and evidenced reductions of white matter integrity in patients with hypercortisolism (50). MRI studies also showed hippocampal atrophy (51) and a blunted hypothalamic glucose reactivity in AN patients, but evidence are not conclusive (52, 53).

Interestingly, Støving et al. discussed how MRI studies revealed structural and functional brain changes associated with endocrine alterations in AN (51). Malnutrition and hormonal imbalances, such as disruptions in HPA axis, are linked to brain volume reductions, particularly in gray and white matter. These MRI findings suggest that chronic energy deficiency and chronic hormonal dysregulation may lead to brain atrophy, affecting regions involved in appetite regulation, cognition, and emotional processing (51). Similarly, Tolle et al. evidenced that functional MRI studies have shown altered activity in the hypothalamus and other brain areas involved in appetite regulation and stress response. The authors suggest that these functional changes may underlie some of the behavioral symptoms of AN, such as altered appetite perception and emotional dysregulation (52).

Thus, the main evidence collectively highlight that functional MRI research has begun to reveal alterations in brain activity, which may be central to understanding the neurobiological basis of AN symptoms and future research should focus also on this theme.

### 5.1 HPA hormone levels in AN

In the first studies on this issue, conducted in the late 1970s and early 1980s, daily mean cortisol concentrations were elevated in up to 80% of women with AN (45, 46). These results were then confirmed in subsequent studies, in which also elevations in urine-free cortisol levels, late night salivary cortisol levels, morning serum cortisol levels and cortisol binding globulin (CBG) were showed (6, 47, 54–56). Different techniques are currently available for measuring cortisol, but it's important to underline that in the measurements of this hormone there are limitations related to the diurnal variation of cortisol and/or of CBG. A highly accurate measurement method is the 24 h pooled serum cortisol, but it is challenging and time-consuming; another way to obtain daily measures of cortisol is the 24 h urinary cortisol method, can be useful in cases where CBG levels may be affected by other factors (as use of exogenous estrogen) (6, 57). Free cortisol is usually directly measured or calculated from total cortisol and CBG using the Coolens equation (58). Interestingly, in malnourished children a reduction in CBG has been shown, correlated to a rise in “free cortisol” levels (59), but these data were not confirmed in AN patients.

Cortisol levels in AN patients were also studied upon low-dose dexamethasone suppression testing (DST) (morning cortisol measured after oral administration of 1 mg of dexamethasone the evening before): indeed, in studies evaluating AN patients and controls, it was reported that upon low-dose DST, the mean serum cortisol level was significantly higher in AN compared with controls. No data are available for high dose DST in these patients (6).

The elevation in serum cortisol levels in AN was first reported by Landon and colleagues in 1966 (60). Later, Boyar et al. evidenced that the 24 h mean serum cortisol levels were nearly doubled in AN (45). The authors hypothesized that this condition was related to a longer half-life due to a decreased metabolic clearance of cortisol (6, 45).

Currently, the cause of HPA hyper-activation has not been cleared yet. Some authors stated that hypercortisolism in AN is due to changes in cortisol half-life and/or to a reduction of its clearance metabolic rate (45, 61–63). However, other authors suggested that hypercortisolism in these patients is due to primary neuro-endocrine abnormalities in the control of HPA (61, 63–65). Indeed, elevated CRH levels have been shown in cerebrospinal fluid of AN patients (65, 66) and cortisol resistance to DST is common in patients with AN, suggesting an impaired sensitivity to the glucocorticoid feedback action (67–71). The state of CRH hypersecretion in AN patients is also supported by the increase of ACTH together with high levels of cortisol, correlated to increased ghrelin levels and protracted nutritional deprivation.

This is further sustained by normal cortisol response to stimulation with ACTH (61, 70) but a weakened response to stimulation with CRH (51).

Interestingly, Lanfranco et al. (61), aiming to define the adrenal sensitivity to ACTH in AN, evaluated cortisol, aldosterone and DHEA responses to the sequential administration of low and supramaximal ACTH doses in 10 young women affected by AN. Then, the authors compared the results obtained to those recorded in 10 healthy controls The results showed no significant differences in cortisol and DHEA responses to ACTH doses in AN patients and controls, evidencing that the sensitivity to ACTH of the adrenal gland is preserved in anorexia nervosa.

The hypercortisolemia proper of these patients have also been correlated, in addition to the stress of prolonged nutritional deprivation (47), to increased ghrelin levels, that stimulate CRH (72–74). CRH is anorexigenic and it might contribute to the severity of weight loss in these patients (75).

Moreover, it has been hypothesized that hypercortisolaemia works as a means of maintaining euglycaemia in AN patients (47).

Importantly, dysregulation of the HPA axis tend to persist in AN even after weight gain, underlining that, despite weight gain, recovery from AN is not complete from the endocrinological point of view (4).

Regarding differences in cortisol levels between the sexes, some studies indicate that differences in the response to neurobiological stress favor men (76, 77), but there have been many reports demonstrating the opposite effect or no relevant differences between the sexes (48, 78, 79). Currently, studies evaluating differences in cortisol levels among different forms of AN are lacking; literature is scarce and not conclusive concerning cortisol levels in other eating disorders (48).

Recently, Thavaraputta et al. performed a systematic review and meta-analysis to study adrenocortical hormones, including DHEA, DHEA-sulphate (DHEA-S), progesterone, 17-hydroxyprogesterone, pregnenolone, cortisol, aldosterone, androstenedione, and testosterone (T) in AN (6). The authors evaluated 101 studies with over 2,500 females with AN and confirmed that, compared to normal-weight controls, AN patients had significantly higher mean levels of cortisol, measured in different ways, as morning cortisol, 12 h and 24 h pooled serum cortisol, 24 h urine cortisol, and after an overnight dexamethasone suppression test (6). In contrast, mean serum total T and DHEA-S levels were significantly lower in AN women with respect to controls (80–82).

These endocrinological disturbances have various deleterious effects on organism (48, 80–82). Specifically, hypercortisolaemia in women and adolescents with AN has been associated with decreased BMD (56, 67), reduced calcium absorption in the gut in addition to an increase in urine calcium excretion (83–85), diminished osteoblast proliferation and decreased synthesis of insulin like growth factor 1 (IGF-1) in osteoblasts, contributing to decreased bone formation (86, 87). In addition, elevated cortisol was also shown to promote an increase in the release of free fatty acids due to lipolysis and lower activity of lipoprotein lipase, leading to an increase in insulin resistance and hyperglycemia (48, 88).

Furthermore, the impact of hypercortisolism on the adaptive thyroid response has also been documented (47). In AN, total T3 levels are often low, likely as an adaptive mechanism to reduce resting energy expenditure and conserve energy for essential functions. These T3 levels are correlated with lower BMI and leptin levels, as well as elevated cortisol and ghrelin levels (47).

Decreased DHEA-S levels have been found also in other states of physiologic stress and critical illness (89, 90). The mechanism responsible for this phenomenon and for the decreased conversion of DHEA to DHEA-S during major stress has not been cleared yet (6, 89, 90).

A way through which higher cortisol levels cause low BMD is the reduction in gonadotropin secretion, that potentially contribute to hypogonadotropic hypogonadism, with subsequent estrogen deficiency and bone demineralization (91, 92). In addition, hypercortisolemia has been associated reduced muscle and extremity lean mass (93). Specifically, Misra et al. performed a study enrolling 23 adolescent girls with AN and 20 healthy girls and examined regional body composition. The authors evaluated levels of estradiol, IGF-1, growth hormone (GH) and cortisol in order to find hormonal determinants of regional body composition in AN adolescent girls and controls (93) and concluded that high levels of GH and cortisol have contrasting associations with fat mass. Specifically, GH favors a redistribution of body fat such that trunk to extremity fat ratio decreases, instead hypercortisolemia levels predict a redistribution of lean body mass such that extremity lean mass decreases (93).

### 5.2 High cortisol levels and metabolic consequences

While AN and Cushing's syndrome are two distinct diseases with markedly different phenotypes, a closer examination reveals some overlap in the clinical manifestations of hypercortisolemia. In both conditions, hypercortisolemia in women is inversely related to BMD and positively correlated with the severity of depression and anxiety symptoms (4). Additionally, hypercortisolemia may suppress the HPG axis, and elevated cortisol levels negatively impact muscle tissue (56, 67). This results in reduced extremity lean mass in both Cushing's syndrome and women with AN (56). Although severe caloric restriction in AN prevents fat accumulation, high baseline cortisol levels during weight restoration are linked to increased trunk fat, similar to what is observed in Cushing's syndrome (4, 67).

On the contrary, signs and symptoms of AN tend to overlap to those of adrenal insufficiency (AI). Indeed, as AI progresses insidiously from the clinical point of view, when evaluating a patients for AN, adrenal insufficiency must be considered in the differential diagnosis in order not to delay adequate treatments (94, 95). Literature is instead scarce concerning the real risk of AI during acute illness in patients with AN.

A relation between high cortisol levels and metabolic syndrome (MetS) in the context of eating disorders has been reported. Indeed, high baseline cortisol levels during AN state, recovery and weight restoration are associated with trunk fat accumulation, similar to patients with Cushing syndrome and/or metabolic syndrome (MetS), a group of risk factors for diabetes and cardiovascular diseases including abdominal fat, high blood pressure, high blood sugar, and hyperlipidemia (96). Interestingly, Yu et al. recently reviewed the comorbid metabolic diseases occurring in patients with eating disorders (97). The authors evidenced a bi-directional relation between MetS and eating disorders: indeed, adolescents with metabolic syndrome were twice as likely to have abnormalities in eating behavior, e.g., restrictive eating or emotional eating than in patients without metabolic syndrome (97, 98).

Moreover, Hudson et al. evaluated the association of the three major eating disorders—AN, bulimia nervosa, and binge-eating disorder—with MetS, or with individual components of metabolic syndrome, such as obesity, type 2 diabetes, hypertension, and dyslipidemia (99).

Current evidence suggests that, although associated to hypercortisolism and increased blood lipid levels, AN confers no excess risk of metabolic syndrome and may be associated with lower risk of certain metabolic syndrome components, as obesity and type 2 diabetes (99). Indeed, the hyperlipidemia reported to be increased in acutely ill patients with AN is not associated with additional risk of cardiovascular diseases and/or metabolic syndrome (99, 100). In addition, although hypercortisolism contributes to glucose intolerance and to reduced insulin sensitivity (101), the risk of type 2 diabetes appears reduced in AN, as Ji et al. reported in a case-registry study of 17,135 patients with AN from Swedish hospitals between 1964 and 2010 (102). The authors found that patients with AN showed a significantly lower incidence of type 2 diabetes with respect to general population (99, 102).

Furthermore, given the role of hormonal functions such as thyroid function in metabolism (103). The interplay between HPA axis disturbances, other endocrine disorders (47), and metabolic impairment cannot be excluded in AN.

### 5.3 Hormone levels and psychological consequences of variations

Hypercortisolemia has been also associated to increased severity of depression and anxiety and poorer cognitive performance (48, 67, 104). More in depth, Lawson et al. recently investigated whether hypercortisolemia is associated with mood disturbance in women with hypothalamic amenorrhea (HA) and AN (67). The authors conducted a cross-sectional study and evaluated 52 women (21 healthy controls, 13 normal-weight women with functional HA, and 18 amenorrheic women with AN) measuring serum samples every 20 min for 12 h overnight and pooled for average cortisol levels (67). The authors also administered to the subjects the Hamilton Rating Scales for Anxiety (HAM-A) and Depression (HAM-D) and found out that AN patients had higher scores in the HAM-D and HAM-A with respect to HA, which, in turn, had higher scores in respect to controls. In addition cortisol levels were highest in AN, intermediate in HA, and lowest in controls (67). In conclusion, hypercortisolemia has been considered a potential mediator of mood disturbance (67).

Interestingly, the same authors also correlated high cortisol levels with lower fasting homeostatic and hedonic appetite, independent of BMI and depressive symptoms (105). Specifically, Lawson et al. recently performed a cross-sectional study evaluating 36 women (13 AN, ten weight-recovered AN and 13 healthy controls) and measured peripheral cortisol and ACTH levels in a fasting state and 30, 60, and 120 min after a standardized mixed meal (105). Then, the authors used a visual analog to assess homeostatic and hedonic appetite and showed that cortisol and fasting appetite were inversely correlated (105).

In addition to cortisol and other androgens, the adrenal cortex is also an important source of testosterone production (106). Indeed, also total testosterone levels have been shown to be lower in AN compared with controls (6, 81). Interestingly, low T levels in these patients have been associated to both low bone mineral density and symptoms of depression/anxiety (81, 107) but, the clinical relevance of T in these patients remains an open question.

## 6 Hypothalamic–pituitary–adrenal axis in AN: therapeutic options

Even if hypercortisolemia in these patients has important adverse effects, no pharmacological approaches are currently recommended to address this condition in AN, especially considering that therapies that lower cortisol might result in AI and/or weight loss, but there is evidence that many psychotropic medications (antipsychotics, antidepressants, and stimulants) affect HPA function (108). Indeed, a recent review by Subramanian et al. reported that antidepressants are associated with a reduction in both basal and post-dexamethasone-CRH (DEX/CRH) cortisol in patients with psychiatric disorders, although some report no change (108).

DHEA supplementation has been studied in AN adolescents as it could have some positive effect in maintaining bone strength, although the overall benefits of this treatment have not been established (82, 109, 110). More in details, Lin et al. recently performed a systematic review and meta-analysis to determine the status of DHEA in AN women and to assess the efficacy of DHEA supplementation for bone health (82). The authors concluded that the combination of DHEA and conjugated oral contraceptives led to increased bone strength and decreased bone loss in older adolescents and adults with closed physes, while this approach was found to have potential detrimental effects on BMD in younger adolescents with open physes (82).

In addition, although total testosterone levels have been shown to be lower in AN and associated to both low bone mineral density and symptoms of depression/anxiety (81, 107), T replacement therapy did not improve BMD in AN women (111), nor the other symptoms of psychological distress (112).

Other treatments as somatostatin administration or cortisol inhibiting drugs have been reported, with limited or even counterproductive effect, but evidence is not conclusive and studies lack on these options (4, 113).

## 7 Limitations and suggestions for future research

There are several limitations that researchers face when attempting to comprehend the correlation between AN, HPA balance and the consequences of HPA dysfunction. Some of these limitations include:

– Lack of long-term studies: the majority of studies evaluating hormonal balance in AN patients are short-term. Long-term studies are essential to evaluate the consequence of HPA dysfunction in the long term and after a nutritional recovery process.
– Lack of data in clinical trial conducted on adolescents: the majority of studies are conducted on adult women.
– Lack of uniformity among the hormonal evaluations (serum vs. urine, basal vs. upon stimulation, 24 h sample vs. single sample)
– Few therapeutic options tested
– Focusing only on the dysfunction of the HPA axis and its implications, without examining in detail the correlation between other hormonal disorders in AN and the HPA axis.

The methodology of this review has certain limitations, as the search for articles was confined to those published within the last 10 years and only articles in English were considered. Additionally, narrative reviews inherently possess limitations in terms of objectivity, completeness of literature search, and interpretation of findings.

## 8 Conclusions

The literature now available demonstrates that women and girls suffering from AN have higher measures of cortisol and lower levels of androgens as compared to controls. These endocrinological disturbances have deleterious effects on the subjects, both from the physical and from the psychological point of view. Thus, it's fundamental for physicians to consider these aspects when assessing AN patients. Additionally, the interplay between HPA axis disturbances and other hormonal disorders, such as thyroidal impairment, should also be considered in AN endocrine and metabolic complications.

As the mechanisms behind the adrenocortical diseases in ED patients remain an open question and considered that there are no available treatments, further research on this issue is necessary, especially in the pediatric age, when preventive measured could be applied.
